# Advancing radiative transfer analysis accuracy in complex geometries with UCWQ and innovative angular mesh

**DOI:** 10.1016/j.mex.2026.103876

**Published:** 2026-03-19

**Authors:** Taoufik Gassoumi, Rachid Said, Rached Ben Younes

**Affiliations:** a Faculty of Sciences of Gafsa, University of Gafsa, 2112 Gafsa, Tunisia; b Research Unit of Ionized and Reactif Media, IPEIM, Monastir University, Ibn El-Jazzar Street 5019 Monastir, Tunisia; c Laboratory of Technology, Energy, and Innovative Materials (TEMI/LR21ES27), Faculty of Sciences of Gafsa, University of Gafsa, 2112 Gafsa, Tunisia

**Keywords:** Shadowing effect on radiation in astrophysical environments, Complex geometries, Obstructions, Ray effect, Novel angular mesh

## Abstract

Accurate representation of coordinates and the computation of the angular quadrature coefficients μ, ξ, and η in complex geometries are essential for advancing the understanding of astrophysical phenomena and various industrial applications. This paper builds upon prior work titled " Enhanced discrete ordinates approach for mitigating shadowing effects in obstructed gas-filled spaces: Implications for astrophysical and industrial applications", which introduced Uniform Constant Weight Quadrature (UCWQ) to address challenges associated with the discrete ordinates method (DOM). Specifically, this research tackles issues related to computational inefficiency and accuracy loss in complex radiative transfer scenarios.

The innovative Pyramidal Angular Mesh (PAM) exhibits an R-squared value of 0.9965. This high statistical agreement directly translates to increased reliability and underscores the model’s strengths in accurately simulating radiative heat transfer, particularly in complex geometrical configurations. A comparative analysis highlights UCWQ’s superior performance with its PAM design over other quadrature methods, such as S_N_ and T_N_, as well as the Finite Volume Method (FVM). Unique aspects of UCWQ include the unlimited choice of directions afforded by the PAM design. This versatile technique is valuable for studying astrophysical systems and analyzing the radiative effects of greenhouse gases, particularly concerning shadowing and ray effects and obstructions in industrial furnaces.

• Employing a novel Pyramidal Angular Mesh (PAM) design to advance the analysis of complex geometries.
• A head-to-head comparison of UCWQ and FVM in terms of performance and accuracy.
• Presenting comprehensive findings and error metrics to assess performance improvements and validate the method.

## Graphical abstract

**Figure:**
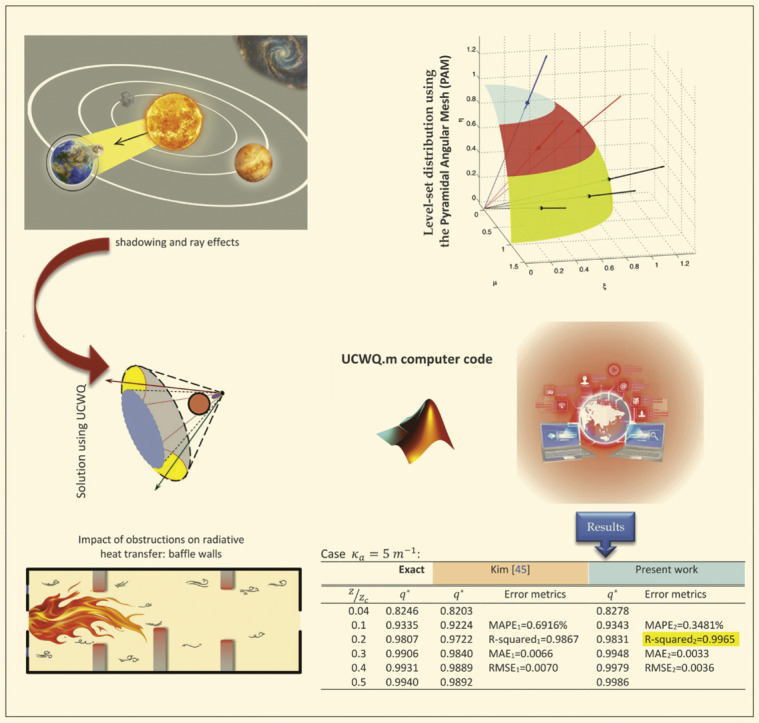

Specifications table

**Table:** 

Subject area	Energy
More specific subject area	Radiative transfer analysis in complex geometries; Computational Fluid Dynamics; Environmental science; Astrophysics
Name of your method	Uniform Constant Weight Quadrature (UCWQ) with new Pyramidal Angular Mesh (PAM)
Name and reference of original method	Enhanced Discrete Ordinates Method (DOM)
Resource availability	Matlab code is provided as supplementary data at: https://ars.els-cdn.com/content/image/1-s2.0-S2590123024009836-mmc1.zip, see appendix in: https://doi.org/10.1016/j.rineng.2024.102728

## Background

The accurate modeling of radiative transfer is crucial across various scientific and engineering fields, particularly in astrophysics [[1], [2], [3]], climate science [4,5], solar energy [6,7], and combustion engineering [[8], [9], [10], [11], [12], [13], [14]]. Traditional methods, such as the Discrete Ordinates Method (DOM) [[15], [16], [17]], while valuable, often present challenges in maintaining accuracy and computational efficiency, especially in complex geometrical scenarios [[18], [19], [20], [21]]. The motivation behind introducing the Uniform Constant Weight Quadrature (UCWQ) method is to provide an innovative approach that effectively addresses these limitations, offering a robust tool for researchers and practitioners alike.

The UCWQ method introduces a new quadrature scheme specifically designed for DOM, which significantly overcomes the shortcomings associated with standard S_N_ and T_N_ quadratures [18,22]. These shortcomings often lead to inaccuracies in temperature distributions and radiative flux profiles, particularly when working with intricate geometries. This is especially evident in the presence of obstructions, which can complicate heat transfer analysis. Recent advancements in numerical solvers and advanced mathematical techniques have been developed to mitigate the effects of obstructions [[23], [24], [25], [26], [27], [28]] and refine the accuracy of thermal models [29,30]. These developments underscore the critical need for robust methodologies that can account for complex geometrical interactions and improve the fidelity of predictive simulations in radiative heat transfer applications [31,32].

The novel pyramidal mesh design enables unlimited angular refinement, ensuring that distortions are minimized and allowing for more precise simulations in varied applications. One of the critical advantages of the UCWQ method is its ability to mitigate shadowing effects [18,33,34], and reduce errors typically caused by ray effects [18,22,[35], [36], [37], [38]], which are prevalent in conventional radiative transfer modeling. By effectively addressing these issues, the UCWQ enhances the reliability of predictions in scenarios with complex interactions, such as combustion studies, atmospheric pollution layers, and the radiative contributions of greenhouse gases. These industrial applications demand both robustness and accuracy, and the UCWQ method is specifically tailored to meet these requirements.

Moreover, the UCWQ method extends its applicability to astrophysical systems, providing a powerful tool for investigating stellar atmospheres and the structures present within the interstellar medium. In these contexts, where the behavior of radiation plays a critical role in understanding physical processes, the accuracy and efficiency offered by the UCWQ method can lead to new insights and advancements in knowledge.

The development of the UCWQ method is grounded in the need for efficient computational models that do not compromise on accuracy. This methodology seeks to enhance accessibility for researchers and engineers with limited computational resources, making advanced radiative transfer analysis more attainable. By streamlining complex calculations and minimizing resource demands, the UCWQ method fosters innovation and exploration in both established and emerging fields.

In summary, the UCWQ method is positioned as a transformative tool for radiative transfer modeling, providing a solution that combines a novel quadrature scheme and an adaptable mesh design. By effectively addressing key challenges such as shadowing and ray effects, the UCWQ method is set to advance research in numerous applications, from industrial processes to astrophysical investigations, paving the way for more accurate and efficient simulations in the future.

## Method details

The radiative transfer equation (RTE) for an absorbing, emitting, and scattering gray medium is formulated in accordance with reference [39]:

(1) $$\left(\Omega .\nabla \right)I\left(r,\Omega \right)=\kappa_{a}\left(r\right)I_{b}\left(r\right)-\beta \left(r\right)I\left(r,\Omega \right)+\frac{\sigma_{s}\left(r\right)}{4\pi }\int_{\Omega^{\prime }=4\pi }\Phi \left(\dot{\Omega }\Omega \right)I\left(r,\Omega^{\prime }\right)d\dot{\Omega }$$

where $\kappa_{a}$, β, and $\sigma_{s}$ are the absorption coefficient, the extinction coefficient, and the scattering coefficient, respectively. For a two-dimensional axisymmetric problem, the spatial derivative term in the radiative transfer equation (RTE) must be modified to account for the cylindrical coordinate system. According to the formulation presented in reference [9], the spatial derivative term in the RTE takes the following form in cylindrical coordinates:

(2) $$\left(\Omega .\nabla \right)I\left(r,\Omega \right)=\frac{\mu }{r}\frac{\partial \left(r.I\right)}{\partial r}-\frac{1}{r}\frac{\partial \left(\xi .I\right)}{\partial \phi }+\eta \frac{\partial I}{\partial z}$$

**Fig. 1:**
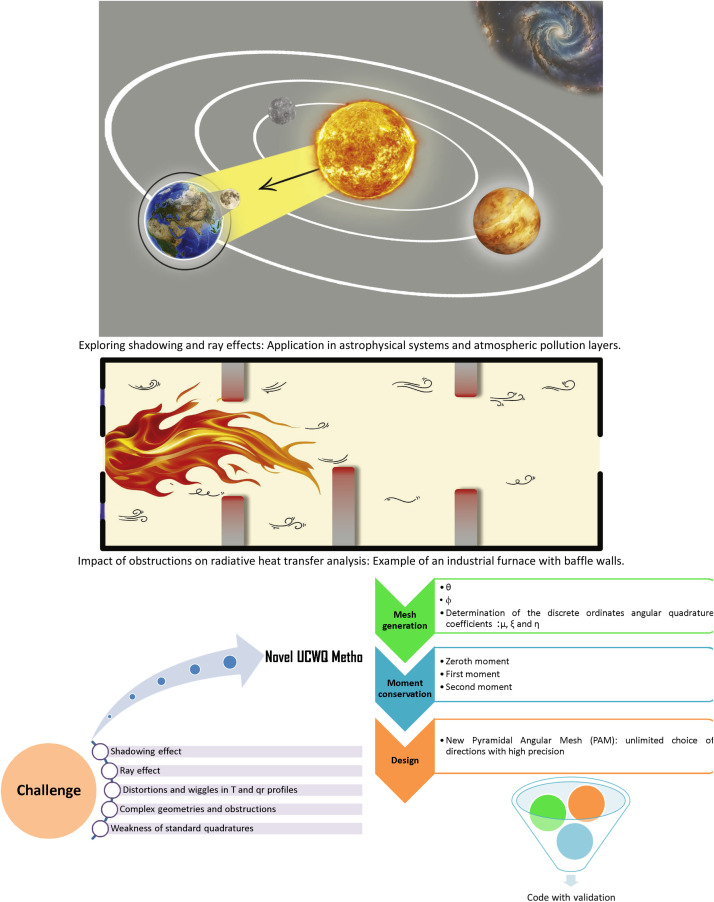
Processes and methodology.

In the context of the radiative transfer equation for a two-dimensional axisymmetric problem, the direction cosines μ=sinθcosϕ, ξ=sinθsinϕ, and η=cosθ are used to represent the direction of radiation propagation along a discrete direction Ω=(μ,ξ,η). These direction cosines are defined with respect to the cylindrical coordinate system (r,z,ϕ), where ϕ is the azimuthal angle. The convergence criterion is achieved when the fluctuations, or "wiggles," observed in the radiative heat flux profile are effectively eliminated [18]. In the case addressed in this paper, where Nd = 20, the convergence criterion during an iterative procedure is:

(3) $$max\left(\left|\frac{I_{P}-I_{p}^{old}}{I_{P}}\right|\right)\leq 10^{-6}$$

with $\left(I_{P}-I_{p}^{old}\right)$ represents the deviation of the radiant intensity at the nodal point P between two consecutive iterations. The system of equations presented in Eq. (4) clearly illustrates that the new method complies with the criteria for the conservation of the zeroth, first, and second moments [40], while also exhibiting symmetry with respect to any axis. The PAM design ensures physical fidelity. Indeed, in the context of radiative transfer, only moments of order (k=0, 1, 2) are associated with distinct physical quantities. More precisely, the zeroth moment corresponds to the incident radiation. The first moment represents the radiative heat flux density, characterizing the directional transport of radiant energy throughout the medium. The second moment is related to the spherical tensor components of the radiation pressure, which are crucial for capturing the momentum transfer induced by radiation. Meanwhile, Eq. (5) summarizes the steps involved in the mesh generation process for the new Pyramidal Angular Mesh (PAM) design. The overall process and methodology described above are illustrated in Fig. 1.

### Moment conservation

(4) $$\begin{array}{c} \left\{ \begin{array}{c} \begin{array}{c} \mathrm{Pyth}\mathrm{agor}\mathrm{ean}\;\mathrm{rela}\mathrm{tion}\mathrm{ship}:\;\mu_{i}^{2}+\xi_{i}^{2}+\eta_{i}^{2}=1\;\;\;\;\;\;\;\;\;\;\;\;\;\;\;\;\;\;\;\;\;\;\;\;\;\;\;\;\;\;\;\;\;\;\;\;\;\;\;\;\;\;\;\;\;\;\;\;\;\;\;\\ {}*\mathrm{Zero}\mathrm{th}\;\mathrm{mome}\mathrm{nt}:\;\sum_{i=1}^{N_{\mathrm{total}}}w_{i}\times \mu_{i}^{0}=\sum_{i=1}^{N_{\mathrm{total}}}w=4\pi \;\;\;\;\;\;\;\;\;\;\;\;\;\;\;\;\;\;\;\;\;\;\;\;\;\;\;\;\;\;\;\;\;\;\;\;\;\;\;\;\;\;\;\;\;\;\;\;\; \end{array}\\ \begin{array}{c} \begin{array}{c} {}*\mathrm{First}\;\mathrm{mome}\mathrm{nt}:\;\sum_{\begin{array}{c} i=1\\ \mu_{i}>0 \end{array}}^{N_{\mathrm{total}}}w_{i}\times \mu_{i}^{1}=w\;\times \sum_{\begin{array}{c} i=1\\ \mu_{i}>0 \end{array}}^{N_{\mathrm{total}}}\mu_{i}=\pi \;\;\;\;\;\;\;\;\;\;\;\;\;\;\;\;\;\;\;\;\;\;\;\;\;\;\;\;\;\;\;\;\;\;\;\;\;\;\;\;\\ \;\mathrm{or}\;\underset{\mathrm{prin}\mathrm{ciple}\;\mathrm{of}\;\mathrm{ener}\mathrm{gy}\;\mathrm{cons}\mathrm{erva}\mathrm{tion}}{\sum_{i=1}^{N_{\mathrm{total}}}w_{i}\times \mu_{i}^{1}=w\;\times \sum_{i=1}^{N_{\mathrm{total}}}\mu_{i}=0}\;\;\;\;\;\;\;\;\;\;\;\;\;\;\;\;\;\;\;\;\;\; \end{array}\;\\ \begin{array}{c} {}*\mathrm{Seco}\mathrm{nd}\;\mathrm{mome}\mathrm{nt}:\;\sum_{i}^{N_{\mathrm{total}}}w_{i}\times \mu_{i}^{2}=w\;\times \sum_{i}^{N_{\mathrm{total}}}\mu_{i}^{2}=\frac{4\pi }{3}\;\;\;\;\;\;\;\;\;\;\;\;\;\;\;\;\;\;\;\;\;\;\;\;\;\;\;\;\;\;\;\;\;\;\;\\ {}*\;k^{\mathrm{th}}\;\mathrm{mome}\mathrm{nt}:\;\int_{4\pi }\Omega^{k}.d\Omega =\sum_{i=1}^{N_{\mathrm{total}}}\mu_{i}^{k}.w_{i}=\left\{ \begin{array}{c} 0\;\forall \;k\;\mathrm{odd}\;\mathrm{inte}\mathrm{ger}\\ 2\pi \times \frac{2}{k+1}\forall \mathrm{keven}\;\mathrm{inte}\mathrm{ger} \end{array}\;\right.\;\;\;\;\;\;\;\; \end{array}\; \end{array} \end{array}\right. \end{array}$$

*PAM design*

(5) $$\begin{array}{c} \left\{ \begin{array}{c} \begin{array}{c} ∴[\begin{array}{c} \theta_{0}=0;\theta_{\mathrm{Nd}}=\frac{\pi }{2}\;\mathrm{and}\;\theta_{i}=\mathrm{acos}\left(1-\frac{2w}{\pi }\frac{i\left(i+1\right)}{2}\right)\;\mathrm{with}\;1\leq i\leq \mathrm{Nd}\;\;\;\;\;\;\;\;\;\;\\ \phi_{1}=\frac{\pi }{4};\;\phi_{i,1}=\frac{\phi_{1}}{i};\;\phi_{i,k}=\phi_{i,k-1}+\frac{\pi }{2\;\times i}\;\mathrm{with}\;2\leq i\leq \mathrm{Nd}\;\mathrm{and}\;2\leq k\leq i\\ \;\phi_{j}=\phi_{i,k}\;\mathrm{with}\;1\leq j\leq N_{T}\;\;\;\;\;\;\;\;\;\;\;\;\;\;\;\;\;\;\;\;\;\;\;\;\;\;\;\;\;\;\;\;\;\;\;\;\;\;\;\;\;\;\;\;\;\;\;\;\;\;\;\;\;\;\;\;\;\;\;\;\;\;\;\;\;\;\;\;\;\;\;\;\;\;\;\;\;\;\;\; \end{array}\;\\ \;\eta_{i}=\cos\theta_{i}\;\;\;\;\;\;\;\;\;\;\;\;\;\;\;\;\;\;\;\;\;\;\;\;\;\;\;\;\;\;\;\;\;\;\;\;\;\;\;\;\;\;\;\;\;\; \end{array}\\ \begin{array}{c} \;\mu_{j}=\sin\theta_{i}\times \cos\phi_{j}=\cos\phi_{j}\times \sqrt{1-\eta_{j}^{2}}\\ \;\xi_{j}=\sin\theta_{i}\times \sin\phi_{j}=\sin\phi_{j}\times \sqrt{1-\eta_{j}^{2}}\;\; \end{array}\;\\ \; \end{array}\right. \end{array}$$

**Fig. 2:**
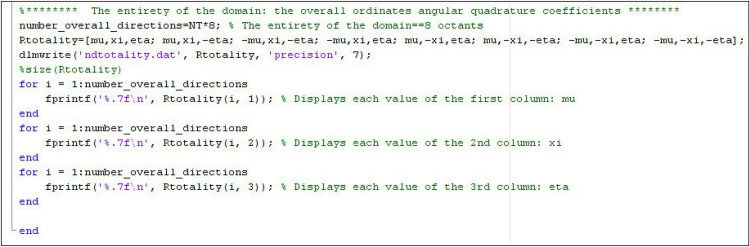
Code instructions (continuation of the UCWQ.m computer code).

The details and methodology followed to develop the new method, UCWQ, are thoroughly described in reference [18]. This method is primarily designed to address issues related to shadowing and ray effects, where the Discrete Ordinates Method (DOM) requires additional refinement to overcome the limitations of traditional quadrature methods such as S_N_ and T_N_ [41]. Gassoumi et al. developed a MATLAB code, provided online as supplementary data with their work [18]. In particular, for cases involving complex geometries, when the geometric configuration lacks symmetry, all coefficients μ, ξ, and η are calculated for all directions, following instructions integrated into the code (see Fig. 2).

**Fig. 3:**
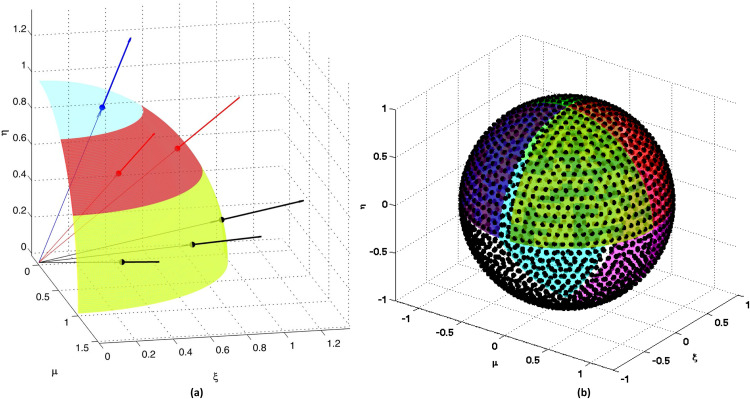
Level-set distribution of angular directions using the pyramidal angular mesh (PAM) for UCWQ quadrature:(a) UCWQ3 per octant, and (b) UCWQ20 with color-coded octants.

As an example, consider the case of UCWQ_3_, which has Nd=3 levels and $N_{T}=6$ directions per octant (see Fig. 3(a)). Therefore, for the entire domain, the total number of directions is given by $N_{total}=8\times N_{T}$, i.e., $N_{total}=4\;\times Nd\times \left(Nd+1\right)=48$ directions knowing that $N_{T}=\frac{Nd\times (Nd+1)}{2}=6$ per octant. In fact, the total number of directions in space is given by 4×Nd×(Nd+1) with Nd=3 representing the quadrature order. If the discrete ordinate $\left(\mu_{i},\xi_{i},\eta_{i}\right)$ is part of the quadrature, then the following discrete ordinates are also included: $\left(\mu_{i},\xi_{i},-\eta_{i}\right)$, $\left(-\mu_{i},\xi_{i},-\eta_{i}\right)$, $\left(-\mu_{i},\xi_{i},\eta_{i}\right)$, $\left(\mu_{i},-\xi_{i},\eta_{i}\right)$, $\left(\mu_{i},-\xi_{i},-\eta_{i}\right)$, $\left(-\mu_{i},-\xi_{i},\eta_{i}\right)$, and $\left(-\mu_{i},-\xi_{i},-\eta_{i}\right)$ (see Table 1). The values presented in Table 1 are consistent with and confirm the moment conservation criteria of the system of equations described in Eq. (4).

**Table 1:** UCWQ3 quadrature sets.

Quadrature order	Ordinates			
	μ	ξ	η	
**UCWQ_3_** **(Nd=3, N_T_=6)** **N_total_=48**	0.2825971 0.6886191 0.2852354 0.9352537 0.6846532 0.2506005	0.2825971 0.2852354 0.6886191 0.2506005 0.6846532 0.9352537	0.9166667 0.6666667 0.6666667 0.2500000 0.2500000 0.2500000	1st octant
	0.2825971 0.6886191 0.2852354 0.9352537 0.6846532 0.2506005	0.2825971 0.2852354 0.6886191 0.2506005 0.6846532 0.9352537	-0.9166667 -0.6666667 -0.6666667 -0.2500000 -0.2500000 -0.2500000	2nd octant
	-0.2825971 -0.6886191 -0.2852354 -0.9352537 -0.6846532 -0.2506005	0.2825971 0.2852354 0.6886191 0.2506005 0.6846532 0.9352537	-0.9166667 -0.6666667 -0.6666667 -0.2500000 -0.2500000 -0.2500000	3rd octant
	-0.2825971 -0.6886191 -0.2852354 -0.9352537 -0.6846532 -0.2506005	0.2825971 0.2852354 0.6886191 0.2506005 0.6846532 0.9352537	0.9166667 0.6666667 0.6666667 0.2500000 0.2500000 0.2500000	4th octant
	0.2825971 0.6886191 0.2852354 0.9352537 0.6846532 0.2506005	-0.2825971 -0.2852354 -0.6886191 -0.2506005 -0.6846532 -0.9352537	0.9166667 0.6666667 0.6666667 0.2500000 0.2500000 0.2500000	5th octant
	0.2825971 0.6886191 0.2852354 0.9352537 0.6846532 0.2506005	-0.2825971 -0.2852354 -0.6886191 -0.2506005 -0.6846532 -0.9352537	-0.9166667 -0.6666667 -0.6666667 -0.2500000 -0.2500000 -0.2500000	6th octant
	-0.2825971 -0.6886191 -0.2852354 -0.9352537 -0.6846532 -0.2506005	-0.2825971 -0.2852354 -0.6886191 -0.2506005 -0.6846532 -0.9352537	0.9166667 0.6666667 0.6666667 0.2500000 0.2500000 0.2500000	7th octant
	-0.2825971 -0.6886191 -0.2852354 -0.9352537 -0.6846532 -0.2506005	-0.2825971 -0.2852354 -0.6886191 -0.2506005 -0.6846532 -0.9352537	-0.9166667 -0.6666667 -0.6666667 -0.2500000 -0.2500000 -0.2500000	8th octant

## Method validation

The performance of UCWQ in the context of spherical geometry with obstructions is examined [18]. Additionally, this paper proposes a comparison between the new UCWQ method and the Finite Volume Method (FVM) [[42], [43], [44]]. For cylindrical geometry, particularly in industrial furnace applications, the work of Kim [45] serves as a reference. The cylindrical furnace has a radius of $r_{c}=1m$ and a height of $z_{c}=2m$, with blackbody side wall $\left(emissivity:\varepsilon_{w}=1\right)$ at a temperature of $T_{w}=0K$. The spatial grid system used consists of $\left(N_{r}\times N_{z}\right)=\left(25\;\times \;51\right)$. The angular mesh grid is defined as UCWQ_Nd_, where Nd = 20, N_T_=210 and N_total_=1680, as shown in Fig. 3(b). All discrete directions possess the same weight $w=\frac{\pi }{2\;\times N_{T}}=\frac{\pi }{2\;\times \;210}=0.007479983$. Additionally, the FVM uses a grid with dimensions $\left(N_{\theta }\times N_{\emptyset }\right)$ with $N_{\theta }=16\;\text{and}\;N_{\emptyset }=14$.

**Fig. 4:**
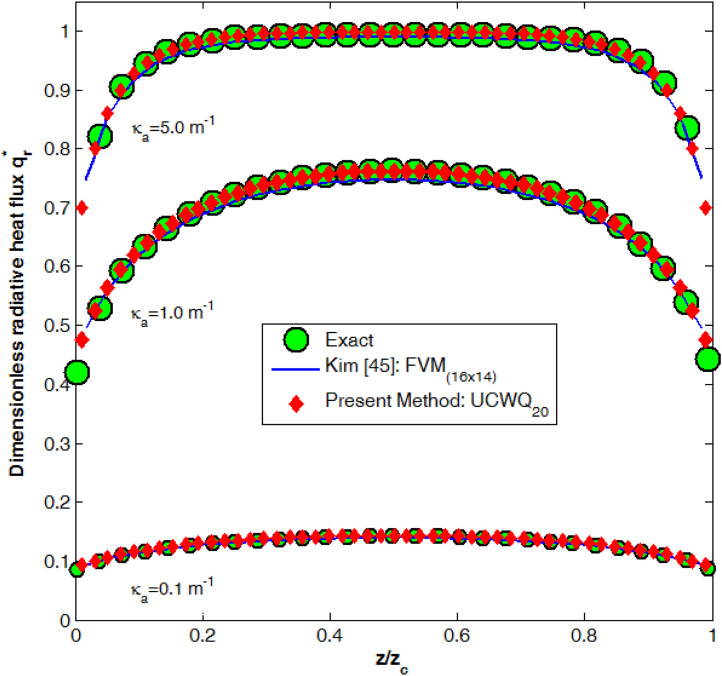
Comparison between UCWQ20 (Ntotal=1680 directions) and FVM $\langle \left(N_{\theta }\times N_{\emptyset }\right)=\left(16\;\times \;14\right)\rangle$ results.

**Fig. 5:**
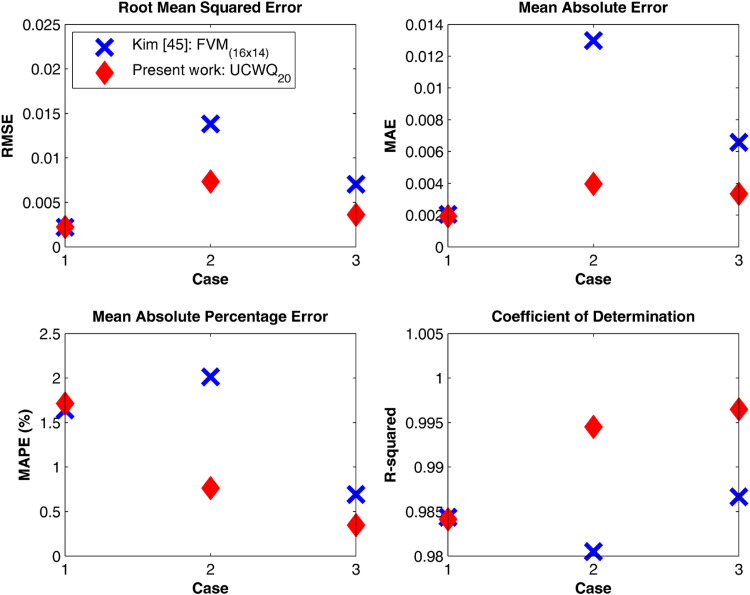
Error metrics analysis: comparison between the FVM and the novel UCWQ method.

Fig. 4 illustrates the dimensionless radiative heat flux for three different values of the absorption coefficient, $\kappa_{a}$, of the gray gas. To assess the accuracy of the new UCWQ method in comparison to the results obtained using the Finite Volumes Method (FVM), a comprehensive error analysis was conducted employing several statistical measures. The Mean Absolute Percentage Error (MAPE) provided a reliable indication of the average accuracy of predictions expressed as a percentage, while the R-squared (Coefficient of determination) evaluated the proportion of variance in the exact data that could be explained by the model, with values approaching 1 denoting high predictive accuracy. Additionally, the Mean Absolute Error (MAE) quantified the average magnitude of errors in the same units as the data, and the Root Mean Square Error (RMSE) assessed the overall performance of the model by giving greater weight to larger deviations between predicted and actual values. Table 2 effectively illustrate the robustness of the newly developed method in comparison to the FVM. The outcomes of this analysis are visually represented in Fig. 5, illustrating the performance of the UCWQ method relative to that of the FVM Table 2.

**Table 2:** Dimensionless radiative heat flux on the side wall, $q^{*}$.

Case 1: $\kappa_{a}=0.1\;m^{-1}$.					
	Exact	Kim [45]		Present work	
z/zzczc	$q^{*}$	$q^{*}$	Error metrics	$q^{*}$	Error metrics
0.02	0.0926	0.0950		0.0968	
0.1	0.1146	0.1138	MAPE_1_=1.6411%	0.1167	MAPE_2_=1.7141%
0.2	0.1285	0.1273	R-squared_1_=0.9844	0.1304	R-squared_2_=0.9841
0.3	0.1366	0.1345	MAE_1_=0.0020	0.1382	MAE_2_=0.0019
0.4	0.1415	0.1382	RMSE_1_=0.0022	0.1423	RMSE_2_=0.0022
0.5	0.1426	0.1401		0.1436	

**Case 2:**$\kappa_{a}=1\;m^{-1}$.					
	Exact	Kim [45]		Present work	
z/zzczc	$q^{*}$	$q^{*}$	Error metrics	$q^{*}$	Error metrics
0.02	0.4818	0.5019		0.4994	
0.1	0.6240	0.6198	MAPE_1_=2.0134%	0.6274	MAPE_2_=0.7627%
0.2	0.7008	0.6893	R-squared_1_=0.9805	0.7014	R-squared_2_=0.9945
0.3	0.7380	0.7248	MAE_1_=0.0130	0.7387	MAE_2_=0.0040
0.4	0.7558	0.7418	RMSE_1_=0.0138	0.7568	RMSE_2_=0.0073
0.5	0.7628	0.7479		0.7623	

**Case 3:**$\kappa_{a}=5\;m^{-1}$.					
	Exact	Kim [45]		Present work	
z/zzczc	$q^{*}$	$q^{*}$	Error metrics	$q^{*}$	Error metrics
0.04	0.8246	0.8203		0.8278	
0.1	0.9335	0.9224	MAPE_1_=0.6916%	0.9343	MAPE_2_=0.3481%
0.2	0.9807	0.9722	R-squared_1_=0.9867	0.9831	R-squared_2_=0.9965
0.3	0.9906	0.9840	MAE_1_=0.0066	0.9948	MAE_2_=0.0033
0.4	0.9931	0.9889	RMSE_1_=0.0070	0.9979	RMSE_2_=0.0036
0.5	0.9940	0.9892		0.9986

**Fig. 6:**
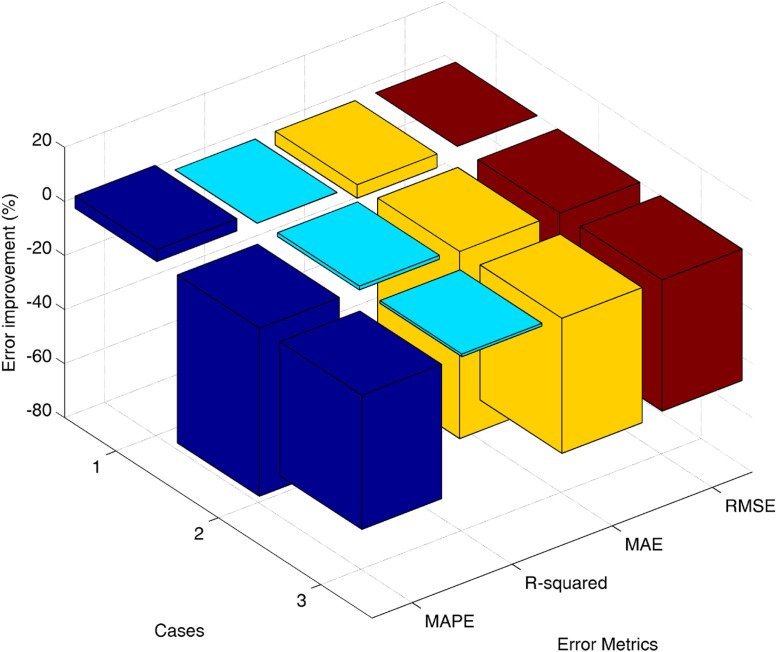
Percentage improvement in Error Metrics: UCWQ20 compared to $\text{FV}M_{\left(16\;\times \;14\right)}$.

Examining the R-squared (R²) values, the results indicate a general improvement for the new Uniform Constant Weight Quadrature (UCWQ) method across all scenarios. The increases in R² values, with R² = 0.9965, reflect a stronger explanatory power of the current method, UCWQ_20_, which achieved higher goodness-of-fit measures than the Kim method ($FVM_{\left(16\;\times \;14\right)}$). As illustrated in Fig. 6, the analysis of the Mean Absolute Error (MAE) shows that the present method also exhibits enhanced performance, yielding error reductions of up to 69.2% relative to the Kim method. Such reductions signify that the new method generates predictions closer to the actual values, which is crucial for overall model accuracy.

The analysis of the Root Mean Square Error (RMSE) highlighted notable improvements in the second and third scenarios for the UCWQ method, with reductions in prediction error of approximately 47.6% and 48.0%, respectively, compared to the FVM method. The performance in the first scenario demonstrated no change, suggesting a degree of robustness in the model's performance. This stability in RMSE measurements is essential, as it indicates reliability in predictions across different instances.

In summary, the UCWQ_20_ method exhibits significant improvements in error metrics relative to the Kim method ($FVM_{\left(16\;\times \;14\right)}$). These findings suggest that the current method, utilizing the new angular pyramidal mesh design, may provide a more precise representation of the underlying data, which is vital for effective predictive modeling. As absorption characteristics vary in the medium, the model's ability to explain more variance (higher R² value) indicates its effectiveness in capturing complex gas behaviors and interactions with thermal radiation at high temperatures. The results confirm that as absorption coefficients increase, the model’s performance improves, underscoring the importance of understanding gas properties for reliable predictive modeling. Overall, these results are crucial for optimizing operational processes in industrial applications, thereby enhancing safety, efficiency, and emissions management.

## Conclusion

The Uniform Constant Weight Quadrature (UCWQ) method represents a significant advancement in radiative transfer modeling within complex geometries. This study demonstrates that UCWQ outperforms traditional quadrature schemes and methods, particularly the Finite Volume Method (FVM), in both computational efficiency and predictive accuracy. Validation against various scenarios reveals that UCWQ effectively addresses challenges such as shadowing and ray effects, which have historically constrained conventional models. The novel Pyramidal Angular Mesh (PAM) design of UCWQ enhances angular refinement, enabling precise simulations applicable across various fields, including combustion processes, greenhouse gas radiation within atmospheric pollution layers, and astrophysics. Comparative error metrics further validate the robustness of UCWQ, demonstrating its capability to deliver accurate radiative flux profiles in complex geometric configurations.

In light of these advancements, several future research directions are proposed. First, expanding the application of UCWQ and PAM to a range of astrophysical environments could yield valuable insights into radiative processes across diverse cosmic structures. Additionally, investigating more complex geometric configurations and various types of obstructions, such as those encountered in industrial furnaces with baffle walls, will deepen our understanding of their impacts on radiation transport. Furthermore, exploring advanced computational methods, particularly the integration of machine learning, has the potential to significantly enhance the predictive accuracy of the UCWQ method.

## Limitations

*The numerical cost is proportional to the complexity of the geometric configuration and the chosen angular resolution. However, the advantages of using UCWQ with PAM design become particularly significant for geometric configurations with shadowing effects, where its benefits are especially pronounced.*

## Ethics statements

**CRediT author statement**

**Taoufik Gassoumi:** Methodology, Formal analysis, Writing – review & editing, Writing – original draft, Visualization, Validation, Supervision, Software, Investigation, Data curation, Conceptualization. **Rachid Said:** Visualization, Investigation, Validation, Supervision, Software. **Rached Ben Younes:** Supervision, Resources, Project administration.

**Supplementary material and/or additional information**

Matlab code: https://ars.els-cdn.com/content/image/1-s2.0-S2590123024009836-mmc1.zip in https://doi.org/10.1016/j.rineng.2024.102728

**Related research article**

*Enhanced discrete ordinates approach for mitigating shadowing effects in obstructed gas-filled spaces: Implications for astrophysical and industrial applications* *☆*

For a published article:

*T. Gassoumi, U.F. Alqsair, R. Said, Enhanced discrete ordinates approach for mitigating shadowing effects in obstructed gas-filled spaces: Implications for astrophysical and industrial applications, Results in Engineering, 23, (2024),102728.* https://doi.org/10.1016/j.rineng.2024.102728

## Declaration of competing interest

Please tick the appropriate statement below (please do not delete either statement) and declare any financial interests/personal relationships which may affect your work in the box below. The authors declare that they have no known competing financial interests or personal relationships that could have appeared to influence the work reported in this paper.

## Data Availability

Data will be made available on request.

## Appendix Supplementary materials

Image, application 1
